# Covid-19 pandemic impact on maternal and child health services access in Nampula, Mozambique: a mixed methods research

**DOI:** 10.1186/s12913-021-06878-3

**Published:** 2021-08-23

**Authors:** Paulo Henrique das Neves Martins Pires, Cynthia Macaringue, Ahmed Abdirazak, Jaibo Rassul Mucufo, Martins Abudo Mupueleque, David Zakus, Ronald Siemens, Celso Fernando Belo

**Affiliations:** 1 Faculty oh Health Sciences, Lúrio University, Nampula, Mozambique; 2 University Mussa Bin Bique, Nampula, Mozambique; 3 Ryerson University, Toronto, Canada; 4 Faculty of Medicine, University of Saskatchewan, Saskatoon, Canada

**Keywords:** Adolescent health, Children health, Covid-19, Health services access, Maternal health, Mozambique, Nampula

## Abstract

**Background:**

The Covid-19 pandemic has so far infected more than 30 million people in the world, having major impact on global health with collateral damage. In Mozambique, a public state of emergency was declared at the end of March 2020. This has limited people’s movements and reduced public services, leading to a decrease in the number of people accessing health care facilities. An implementation research project, The Alert Community for a Prepared Hospital, has been promoting access to maternal and child health care, in Natikiri, Nampula, for the last four years. Nampula has the second highest incidence of Covid-19. The purpose of this study is to assess the impact of Covid-19 pandemic Government restrictions on access to maternal and child healthcare services. We compared health centres in Nampula city with healthcare centres in our research catchment area. We wanted to see if our previous research interventions have led to a more resilient response from the community.

**Methods:**

Mixed-methods research, descriptive, cross-sectional, retrospective, using a review of patient visit documentation. We compared maternal and child health care unit statistical indicators from March–May 2019 to the same time-period in 2020. We tested for significant changes in access to maternal and child health services, using KrushKall Wallis, One-way Anova and mean and standard deviation tests.

We compared interviews with health professionals, traditional birth attendants and patients in the two areas. We gathered data from a comparable city health centre and the main city referral hospital. The Marrere health centre and Marrere General Hospital were the two Alert Community for a Prepared Hospital intervention sites.

**Results:**

Comparing 2019 quantitative maternal health services access indicators with those from 2020, showed decreases in most important indicators: family planning visits and elective C-sections dropped 28%; first antenatal visit occurring in the first trimester dropped 26%; hospital deliveries dropped a statistically significant 4% *(p* = 0.046), while home deliveries rose 74%; children vaccinated down 20%.

**Conclusion:**

Our results demonstrated the negative collateral effects of Covid-19 pandemic Government restrictions, on access to maternal and child healthcare services, and highlighted the need to improve the health information system in Mozambique.

**Supplementary Information:**

The online version contains supplementary material available at 10.1186/s12913-021-06878-3.

## Background

Covid-19 virus pandemic has infected more than 30 million persons in the world (August 2020) [1], and continues to overload the national health care services in most countries [2]. Attempts to control the pandemic have had the secondary effect of limiting access to, and delivery of, public and private health care services [3], with severe unexpected collateral damage [4].

In Mozambique, the President declared the state of emergency at the end of March 2020, when there were no positive cases in the country. This limited travel, reduced all public services and gatherings. A national media information and education campaign, teaching preventive measures, was broadcast on television and radio across the country. These preventive measures might have reduced the number of infections: at the writing of this paper, Mozambique is 9th in the number of cases in Africa (3651). These have occurred mostly in men (60%), mainly within the 25–44-year-old age group (53%); 58% of patients were asymptomatic, 34% had mild symptoms and there were 21 deaths (0,6% death rate, the 7th smallest in Africa). Unfortunately, the preventive measures have led to diminished numbers of health care workers (HCWs) at primary care health centres and hospitals. The pandemic preventive measures aggravated by misinformation through social media networks [5], decreased the number of users at all health care levels. The effects on the national health programs are not well known but the negative impacts are documented in a British National Health System research paper, recommending HCWs and local authorities, pay close attention to prevent health care access restrictions [6].

Many women and children in Mozambique faced barriers as they tried to access health services, even before the COVID-19 pandemic began. Maternal and child health (MCH) is one of the Mozambican government priorities. Interventions have included: implementing family planning (FP), ante-natal assistance, in-hospital deliveries, and childhood monitoring and vaccination programs. These target groups have high morbidity and mortality rates, far below the Sustainable Development Goals and now are at higher risk of worse outcomes given the national response to Covid-19 [7].

Over the last four years, the Alert Community for a Prepared Hospital implementation research (ACPH) project, a partnership between the Faculty of Health Science (FHS) of Lúrio University (UniLúrio) with the University of Saskatchewan (Canada) and Nampula Provincial Health Board (NPHB), has been promoting access to maternal, child and adolescent health care, at Marrere General Hospital (MGH) and Marrere Health Centre (MHC), in Natikiri, Nampula [8]. A base line study in 2016 helped to inform the design of seven strategies. These included interventions to inform local communities, leaders, and traditional birth attendants (TBAs), about sexual and reproductive health (SRH), and how to take an active role in improving their community health services. HCWs received training on emergency obstetrical care, new-born resuscitation, family centred care and obstetric ultrasound and MGH had a new operating room. A motorcycle ambulance system was developed to improve access for pregnant women [ 9].

Nampula is the third largest city in the country (660,000 people), and capital of the most populated province (6 million) in Mozambique. It has the third highest Covid-19 incidence in the country (517 cases), after Maputo and Cabo Delgado. The number of Covid-19 cases is insignificant when compared to malaria, cholera, tuberculosis, human immunodeficiency virus (HIV) infections or road accidents deaths.

Given this, we planned to evaluate the impact of the state of emergency governmental preventative measures for Covid-19, and Ministry of Health (MH) priority decisions, on access to MCH care services, and the resilience of health care units (HUs) to the pandemic. We also want to access the effect of ACPH interventions in mitigating some of the secondary effects of Covid-19 preventive strategies in Natikiri, our intervention area, comparing with two representative care facilities in Nampula city.

## Methods

We aimed to assess the access to maternal child healthcare units under the state of emergency due to Covid-19. We used a mixed methods research, descriptive, cross-sectional, and retrospective, using a review of patient visit documentation. We compared maternal and child health care unit statistical indicators from March–May 2019 to the same time-period in 2020. This was then followed by interviews (the interview guide was developed for this study, see Appendices) of MCH HCWs, TBAs and clients using the system.

Quantitative data was obtained from health care monthly official statistics and the MCH statistics department in the HUs.

We gathered data from a comparative city health centre (25 de Setembro) and the main city referral hospital (Nampula Central). The MHC and MGH were the two ACPH intervention sites.

Quantitative data was recorded on Microsoft Office Excel, double checked by research assistant and statistics professor, then analysed by number and percentage. SPSS21 software was used to test for significant changes in access to MCH services between 2019 and 2020, using KrushKall Wallis, One-way Anova and mean and standard deviation tests.

ACPH had a data base with phone numbers of TBAs in the Natikiri area. They were interviewed and in collaboration with them, we were able to find mothers and pregnant women willing to undergo an interview. HCWs were interviewed in their workplace. Subjects were a purposive sample of HCWs (nine), TBAs (six in Natikiri) and patients or users (six in Natikiri) of MCH services. Due to the Presidential declaration of a State of Emergency, in response to the COVID-19 pandemic, community members were interviewed by cell phone and HCWs in person respecting preventive measures. A total of 19 female participants were interviewed each for 30 min: six users (mothers and pregnant women), four TBAs from Natikiri district, three MCH nurses from MHC, three MCH nurses from HC 25 Setembro, and three from NCH. The interviews were conducted in Portuguese or Emakhuwa (local language), following participant preference, by duly trained research assistants. The interviewers were fluent in the local language, were unknown to the participants, and took written notes. No interviews were repeated.

After receiving verbal consent interviews were recorded and subsequently transcribed to Microsoft Office Word by the same research assistants. Qualitative data was then transferred to NVIVO program for analysis. We used inductive content analysis, targeting four speech categories and nine themes: 1) Knowledge about Covid-19 disorder (cause, symptoms, prevention, social impact); 2) Impact of CoVid-19 on access to health care units (general population and individual client behaviour); 3) Health system response (HCWs MCH); 4) Impact on motorcycle-ambulance usage (demand, service).

This research was allowed by the FHS, NPHB, Nampula City Health Directorate, and approved by UniLúrio Bioethics Health Committee. We followed all Helsinki Declaration (2013) recommendations. All participants were volunteers, anonymity was guaranteed (no data was linked to identification), freedom to withdraw without any negative effect, and recording an informed consent. Recorded and transcript data were protected by a password to grant confidentiality. This research had no risk or remuneration to participants. They agreed to give their time and opinion on this topic to benefit the general population, contribute to improve public health policy interventions and implementation research.

## Results

### Quantitative results

Comparing the three months of Covid-19 pandemic in Natikiri, Mozambique in 2020, with the same period in 2019, concerning MCH services access indicators, data showed an increase in home deliveries (74%), the number of pregnant women attending their first ante-natal visit (19%), the number of women completing four ante-natal visits (19%), and the number of well-baby visits (14%). There were decreases in the number of women attending their first ante-natal visit at less than 12 weeks gestation (26%), elective c-sections (28%), all not statistically significant. There was a statistically significant decrease of 4% in hospital deliveries (*p* = 0,046, see Table 1). In the non-intervention area, data showed an increase of 125% in the number of women with four ante-natal visits, and a decrease of 12% of the number of women with first ante-natal visit in the first trimester, all not statistically significant (see Table 2).

**Table 1:** Maternal health services in intervention area, Natikiri, 2019–2020

MATERNAL HEALTH INTERVENTION AREA												
**MARRERE GENERAL HOSPITAL**	**2019**			**TOTAL**	**M+/−SD**	**2020**			**TOTAL**	**M+/−SD**		
**MARRERE HEALTH CENTRE**	**March**	**April**	**May**			**March**	**April**	**May**			Change	*P*
**Total 1st Ante-natal visits**	158	NDA	200	358	165.33+/− 31.64	142	159	126	427	142.33+/−16.50	19%	0.327^**^
**No. Women with Ante-natal Consultation in 1st trimester**	6	5	20	31	10.33+/−8.38	7	6	10	23	7.67+/−2.08	−26%	0.658^*^
**No. women with 4 Ante-natal Consultations**	48	45	85	178	59.33+/− 22.28	36	123	52	211	70.33+/−46.30	19%	0.730^**^
**No. Post-partum Consultations**	153	155	185	493	199.33+/−67.86	371	5	187	563	108+/− 40.45	14%	0.116^**^
**No. Intra- Hospital Deliveries**	135	111	142	388	156.00+/−25.12	119	130	125	374	126.67+/−5.51	−4%	0.046^*^
**No. Extra-hospital Deliveries**	4	7	8	19	6.33+/−2.08	9	10	14	33	11.00+/−2.66	74%	0.074^**^
**No. Elective C-section**	8	3	7	18	6.67+/−1.53	6	4	3	13	4.33+/−1.53	−28%	0.135^**^
**Maternity – Emergency Room**	152	137	169	458	152.67+/− 16.01	162	160	153	475	158.33+/−4.72*p*	4%	0.588^**^

NDA: data not available

Legend: ^*^ Krus kal Wallis Test; ^**^ One-way ANOVA; M+/−SD – Mean +/− Standard deviation

When Krus kal Wallis and One-Way ANOVA tests are run, they showed that there was only statistical significance in number of intra-hospital deliveries between both time periods when *p*-value is 0.046 and α = 0.05

**Table 2:** Maternal health services in non-intervention area, Nampula, 2019–2020

MATERNAL HEALTH NON-INTERVENTION AREA															
**NAMPULA CENTRAL HOSPITAL**	**2019**						**TOTAL**	**2020**						**TOTAL**	
**25 SEPTEMBER HEALTH CENTER**	**NCH**	**25 HC**	**NCH**	**25 HC**	**HCN**	**25 HC**		**NCH**	**25 HC**	**NCH**	**25 HC**	**NCH**	**25 HC**		
	**Mars**		**April**		**May**			**Mars**		**April**		**May**			Change
**Total 1st Ante-natal visits**	NDA	1910	21	1640	NDA	1420	4991	86	1858	21	1597	35	1186	4783	−4%
**No. Women with Pre-natal Consultation’s 1st trimester**	NDA	2804	NDA	2640	NDA	2070	7514	NDA	2710	NDA	2025	NDA	1858	6593	−12%
**No. women with 4 Ante-natal Consultations**	NDA	203	NDA	180	NDA	247	630	NDA	197	NDA	236	NDA	982	1415	125%
**No. Post-partum Consultations**	NDA	947	NDA	815	NDA	1033	2795	NDA	836	NDA	1015	NDA	910	2761	−1%
**No. Intra- Hospital Deliveries**	NDA	735	NDA	852	NDA	855	2442	726	735	649	852	624	855	4441	NC
**No. Extra- hospital Deliveries**	NDA	NDA	NDA	NDA	NDA	NDA	0	NDA	NDA	NDA	NDA	NDA	NDA	0	NDA
**No. Elective C-section**	NDA	NA	NDA	NA	NDA	NA	0	302	NA	268	NA	301	NA	871	NDA
**Maternity - ER**	NDA	410	NDA	390	NDA	413	1213	875	397	809	245	741	178	3245	NC

NDA: data not available

NA: not applicable, service does not exist

NC: non comparable

Concerning children and adolescent health services access indicators, quantitative data in the ACPH intervention area showed a decrease in FP visits (28%), number of childhood vaccinations (20%) and children completely vaccinated (18%), all without statistical significance (see Table 3). In non-intervention area we had a decrease of 16% in the number of children completely vaccinated and the number of adolescents and youth visits, all without statistical significance (see Table 4).

**Table 3:** Child and adolescent health services in intervention area, Natikiri, 2019–2020

CHILD AND ADOLESCENT HEALTH INTERVENTION AREA												
**MARRERE HEALTH CENTRE**	**2019**			**TOTAL**	**M+/−SD**	**2020**			**TOTAL**	**M+/−SD**		
	**March**	**April**	**May**			**March**	**April**	**May**			Change	*p*
**Family Planning Consultations**	175	NDA	276	451	164.67+/−20.21	62	124	138	324	187.67+/−18.3	−28%	0.839^**^
**No. Children vaccinated**	156	236	202	594	199.33+/−40.42	180	157	140	477	159.00+/−20.08	−20%	0.197^**^
**No. children with complete vaccination**	105	124	150	379	126.33+/−22.59	172	68	69	309	101.33+/−61.27	−18%	0.544^**^
**Adolescents and Youth Friendly Service visits**	NDA	NDA	NDA	0		NDA	NDA	NDA	0		NDA	

NDA: data not available

Legend: ^*^ Kruskal Wallis Test; ^**^ One-way ANOVA; M+/−SD – Mean +/− Standard deviation

**Table 4:** Child and adolescent health services in non-intervention area, Nampula, 2019–2020

CHILD AND ADOLESCENT HEALTH NON-INTERVENTION AREA															
**NAMPULA CENTRAL HOSPITAL**	**2019**						**TOTAL**	**2029**						**TOTAL**	
**25 SEPTEMBER HEALTH CENTER**	**NCH**	**25 HC**	**NCH**	**25 HC**	**NCH**	**25 HC**		**NCH**	**25 HC**	**NCH**	**25 HC**	**NCH**	**25 HC**		
	**March**		**April**		**May**			**March**		**April**		**May**			Change
**Family Planning Consultations**	NDA	347	314	328	NDA	297	1286	401	315	314	293	234	389	1946	NC
**No. Children vaccinated**	NDA	1593	NDA	1475	NDA	1497	4565	528	1567	494	1376	475	1538	5978	NC
**No. children with complete vaccination**	NA	293	NA	190	NA	193	676	NA	219	NA	141	NA	209	569	−16%
**Adolescents and Youth Friendly Service visits**	NA	228	NA	251	NA	221	700	NA	189	NA	180	NA	218	587	−16%

NDA: data not available

NA: not applicable, service does not exist

### Qualitative results

#### Knowledge of the disease

When evaluating knowledge of the disease, we found that the basics were known by all groups. Users and TBAs were able to mention at least three major symptoms such as cough, fever and difficulty breathing**.**

“ … *it’s a flu, in which the person has a cough, headache, neck pain, feels cold and has fever.*” (TBA, Natikiri).

They were also able to mention simple preventive methods, such as washing hands, social distancing, and wearing masks whenever in public.

“ … *we have to wash our hands with water and soap or ashes*” (Post-partum women, Natikiri).

“ … *we have to use masks, whenever we go out!’*” (Pregnant women, Natikiri).

As expected, HCWs had more knowledge on the origin of the disease,

*“Covid-19 is a contagious disease originated in China and is caused by a new coronavirus SARS-CoV-2”* (MCH Nurse, MHC).

Also, on symptoms, and prevention methods:

“ … *if the person travels to a country contaminated by Covid-19 he has to be quarantined for 14 days”* (MCH Nurse, 25 September HC).

*“ … everyone needs to use masks and maintain social distancing of 1.5m.”* (MCH Nurse, NCH).

#### Impact of Covid-19 in access to health care units

During interviews, all groups stated that they anecdotally saw the number of people frequenting the HUs decreased significantly due to the fear of contamination in the HU.

The TBAs related that there was a reduction in the number of patients seen in the community. Also, both clients and TBAs, mentioned that due to the Covid-19 pandemic, important tasks such as attending churches and mosques was prohibited. Initiation rites were also limited, as they are considered large gatherings. Work overall was affected, as people were forced to stay home. Farming was reduced to occasional days or ceased completely. Additionally, TBAs referred a lower number of their clients’ visits. They mentioned that they also respected and enforced prevention measures, with the few community members who did visit them.

*“The number of health professionals has decreased, and they leave early, so the waiting time has increased a bit”* (TBA, Natikiri).

HCWs noted a much lower workload but also a reduction in MCH HCW’s numbers and their work hours.

*“The flux of patients is reduced; it may be because they fear coming to the hospital thinking that they might be contaminated here in the Nampula Central Hospital”* (MHC Nurse, NCH).

*“ … in the wards there is only one nurse per shift, and because of the pandemic if one gets sick, we will be forced to work every day to cover her!”* (MCH Nurse, MGH).

Interviews with patients showed they recognised behaviour change in the population to prevent the infection, and this was resulting in a reduction in access to health services.

*“ … and I avoid going to the health centre, unless it is really urgent, because of this new infection!”* (Post-partum woman, MHC).

#### Health system response

The lack of HCWs in HUs was seen in all four of centres assessed. This was a recurrent complaint from HCWs, TBAs and patients.

HCWs continue to educate the community regarding Covid-19 prevention methods, and about the necessary conditions for consultations.

“ … *community awareness speeches about prevention methods continue*.” (MCH Nurse, MHC).

“ … *we reinforce measures and make the community understand to comply to the measures of prevention of this disease.*” (MCH Nurse, NCH).

“ … *the health professionals refuse to treat patients with no masks and that didn’t wash their hands!*” (MCH Nurse, NCH).

Clients and TBAs mentioned that they continued to attend healthcare services, mostly to vaccinate their children. This was because the vaccines were no longer available in the communities through mobile brigades’ services, forcing parents to go to the HU to get the child vaccinated.

“ *… these vaccines have not come to the community, so the mothers have to go to the hospital!”* (Post-partum women, Natikiri)*.*

#### Impact on motorcycle-ambulance usage

The motorcycle ambulance system was developed by the ACPH project as an urgent transport plan for pregnant women and other emergencies in the community. The users and TBAs mentioned that the motorcycle ambulances continued to circulate normally in some areas and in others reduced. The nurses in MHC and MGH were not able to provide information on this as they did not interact directly with the system.

*“ … the motorcycle ambulances have reduced their circulation because of the disease!”* (TBA, Natikiri).

*“ … they are not using! Most of them have their own motorcycles.”* (Post-partum women, Natikiri)*.*

When Kruskal Wallis and One-Way ANOVA test were run, they showed no statistical significance between both time periods because the p-value>α > 0.05.

When Kruskal Wallis and One-Way ANOVA tests were run, they showed that there were no statistically significant differences between both time periods when *p*-value is > 0.5.

When Kruskal Wallis and One-Way ANOVA test were run, they showed no statistical significance between both time periods because the p-value>α > 0.05.

## Discussion

Assessment of primary and secondary health care services during this pandemic, was done to determine level of universal health coverage. We assessed health care centres and hospitals, where primary care should be a strong indicator. Government’s Covid-19 pandemic preventive measures might be reducing the number of Covid-19 cases in Mozambique, but they have reduced maternal, children and adolescent’s health services access in Nampula. These facts are recognised globally [10]. It is also known that countries’ primary health care systems strength, does not influence the pandemic mortality rate, which is more dependant of perceived stringent border control, movement restriction, and testing regimes [11].

The preventative measures promoted by the government targeting community health workers, TBAs, traditional leaders and healers through training [12], and the general population using a media campaign launched in May 2020, has led to a reduced access to the public health system, for regular primary and secondary health care services. This might be an opportunity for the MH to reformulate its population information and education strategy [13].

The health information system is deficient like in other countries in Africa [14], and this has limited our conclusions. The NCH has poor quality records and MGH does not incorporate all MHC indicators.

The number of pregnant women with four ante-natal visits increased, but we consider this result independent of Covid-19 impact, since the three previous visits have occurred before the state of emergency.

Comparing the city centre HUs with semi-rural Natikiri, with a higher rate of illiteracy, we saw a slight positive impact of ACPH project in maternal health (number of women in first ante natal visit, women with four ante-natal visits, post-delivery visits, increasing faster than the demographic increase rate of 2.8%) [15]. We did not see any difference in child health indicators (reduction in the number of children coming for vaccination and children completely vaccinated) and adolescent’s health (Adolescent and Youth Friendly Service visits). Results support some ACPH outputs have led to a more informed community response. Our data suggests that Natikiri and Mozambican national public health education interventions, must improve their health care behaviours for children and adolescents, namely encouraging hospital deliveries, vaccination, and contraception [16]. Contraception is an essential need for adolescents and young adult patients, and new approaches should be developed to provide this crucial care [17].

Study limitations: Nampula province has had the highest incidence of Covid-19 positive cases in Mozambique in June 2020 (33%, 293 / 889) [18], and this was one thing that motivated us to carry out this study. MH statistical data is not currently available in several HUs and there are several data collecting issues, limiting our analysis and conclusions. Telephone interviews due to emergency state’ social distancing, led to loss of important communication indicators such as body language. Our study found a low impact of the Covid-19 pandemic governmental preventative measures on MCH care services access, probably since the assessed period included March (state of emergency declared on the 23rd), April and May, the early period of the pandemic.

## Conclusion

Our results demonstrated negative collateral effects of Covid-19 pandemic governmental preventative measures on MCH care in Nampula, reducing the number of maternity deliveries and increasing home births.

ACPH project, informed the local population on SRH, moderately reducing some of these effects, but had no impact on child and adolescent health. This showed the need to improve the health information and education system in Mozambique, targeting preventive interventions with children and youth, namely on FP.

This could be done applying an HCWs training program about communication and values, and designing an innovative media campaign, reaching the rural population using local languages, and community health workers. MH, although developing a media campaign to alert the population to access normal preventive services and chronic diseases consultations, is not reaching the general population, who have low Portuguese language capacity, and few radios and TVs. This will require investing in effective communication with urban and rural populations.

The ACPH project has implemented community-based interventions to improve understanding of SRH, and further research will assess the long-lasting effect of this on reducing the negative collateral effects of Covid-19 pandemic in Natikiri.

## Supplementary Information

**Additional file 1.**

## Data Availability

The authors declare that quantitative and qualitative data supporting the study findings are available when reasonable request is made to the corresponding author (druidatom@gmail.com).

## Abbreviations

ACPH: Alert Community for a Prepared Hospital
AYFS: Adolescent and Youth Friendly Service
Covid-19: SARS-CoV-2 virus infection
FHS: Faculty of Health Sciences
FP: Family planning
HCW: Health care worker
HIV: Human immunodeficiency virus
HU: Health unit
MCH: Maternal and child Health
MGH: Marrere general hospital
MH: Mozambican Ministry of Health
MHC: Marrere health centre
NCH: Nampula central hospital
NCHD: Nampula City Health Directorate
NPHB: Nampula Provincial Health Board
PHC: Primary health care
SRH: Sexual and reproductive health
TBA: Traditional birth attendant
UniLúrio: Lúrio University
