# Supplementary material for: Covid-19 pandemic impact on maternal and child health services access in Nampula, Mozambique: a mixed methods research
Source: BMC Health Serv Res. 2021 Aug 23;21:860. doi: 10.1186/s12913-021-06878-3 (PMC8381138; doi:10.1186/s12913-021-06878-3)
Supplement: Supplementary file 1 — Additional file 1. [file 12913_2021_6878_MOESM1_ESM.docx]

**Name of the project:**

Date of the interview:

Start and End time:

____:____H

____:____H

Duration:

Alert Community for a Prepared Hospital Continuum

**Title of the transcription**

1. **Identification (target group):**
   1. **Name of interviewer:**
   2. **Participant interviewed.**

Number of participants interviewed:

| Code used | Meaning | Language used |
| --- | --- | --- |
|  |  |  |

- - 1. Demographic characteristics of the participant

| Code | Gender | Age | Educational level |
| --- | --- | --- | --- |
|  |  |  |  |

1. **Geographical area and surroundings**

**Generic location:**

**Specific location:**

**Conditions of the location:**

1. **Interview questionnaire**
2. What do you know / understand about Covid-19? In a few words what can you tell about this?
3. Have there been any changes in the community since the beginning of the disease? If yes, which ones?
4. Have you noticed any changes in community behaviour towards the health units services / care in this period (since the State of Emergency declaration)?
5. Have there been any changes in your daily routine? If yes, what? How have you coped with these changes?
6. Have the motorcycle ambulance circulated the same way as before? If not, could you mention why?
7. In your opinion, are there enough healthcare professionals to cover the necessities of maternal and child health services?
8. Have there been any changes in access to services by pregnant and puerperal women? If yes, can you tell me why? Have women continued to deliver babies in the health units 24 hours / 7 days a week?
9. Have women frequented the outpatient consultations? If not, could you explain why?
10. Is the community adhering to the Expanded Vaccination Programme regularly? If not, would you tell us why?
